# Transition to reconstructibility in weakly coupled networks

**DOI:** 10.1371/journal.pone.0186624

**Published:** 2017-10-20

**Authors:** Benedict J. Lünsmann, Christoph Kirst, Marc Timme

**Affiliations:** 1 Network Dynamics, Max Planck Institute for Dynamics and Self-Organization (MPIDS), 37077 Göttingen, Germany; 2 Max Planck Institute for the Physics of Complex Systems (MPIPKS), 01187 Dresden, Germany; 3 Rockefeller University, NY 10065-6399 New York, United States of America; 4 Bernstein Center for Computational Neuroscience (BCCN), 37077 Göttingen, Germany; 5 Chair for Network Dynamics, Center for Advancing Electronics Dresden (cfaed) and Institute for Theoretical Physics, Technical University of Dresden, 01062 Dresden, Germany; 6 Department of Physics, Technical University of Darmstadt, 64289 Darmstadt, Germany; University of Michigan, UNITED STATES

## Abstract

Across scientific disciplines, thresholded pairwise measures of statistical dependence between time series are taken as proxies for the interactions between the dynamical units of a network. Yet such correlation measures often fail to reflect the underlying physical interactions accurately. Here we systematically study the problem of reconstructing direct physical interaction networks from thresholding correlations. We explicate how local common cause and relay structures, heterogeneous in-degrees and non-local structural properties of the network generally hinder reconstructibility. However, in the limit of weak coupling strengths we prove that stationary systems with dynamics close to a given operating point transition to universal reconstructiblity across all network topologies.

## Introduction

Complex networked systems generate dynamics and thus functions that fundamentally depend on how their units interact [1–3]. As a consequence, knowing the interaction topology of such systems is a key towards understanding them [4–12]. Yet, direct access to the topology of physical interactions is largely limited for many natural systems and across scales, ranging from metabolic and gene regulatory networks on the subcellular level to neural circuits of millions of cells, to food webs among organisms and planetary climate networks [10, 13–21]. Thus, measures of pairwise statistical dependencies between time series of the dynamics of their units are often employed as proxies for physical interactions [15–17, 21–27]. Assuming sufficiently many and sufficiently accurate data, each such method provides useful information about how the considered statistical dependency measures vary across pairs of units. The value of such a statistical measure, thresholded as desired, e.g. for significance against coincident correlations, may be taken to quantify the interactions among these units. Yet, such measures themselves do not necessarily provide immediate insights into how the units are directly influencing each other via physical interactions. In particular, what do correlations generally tell us about direct physical interactions in network dynamical systems? And is it possible to detect direct physical interactions among units by thresholding these measures to reconstruct the topology of the network?

Here, we systematically address this question on a conceptual level and identify limits of network reconstructibility based on thresholding pairwise measures of statistical dependence. In general, non-linearities of intrinsic and coupling dynamics, correlated noise sources, heterogeneities in time scales and coupling strengths as well as nontrivial network topology jointly create complex statistical correlation patterns. To reveal principal limits of reconstructibility originating from network interactions (toplogy and strength), we here focus on systems with dynamics around a given operating point. More specifically, we analyze the idealized setting of linearly coupled systems with homogeneous dynamical parameters receiving independent additive noise inputs and evaluate network reconstruction from thresholding linear correlations obtained from sufficiently long time series. Reconstruction of physical interactions generally is at least as hard in any more complex setting, e.g., involving non-linear dynamics and adequate measures of statistical dependence such as mutual information. We explicate limits of reconstructibility due to local common cause structures, local relay structures, topological in-degree heterogeneities as well as non-local structural elements. Despite these limitations our analysis interestingly also reveals that, stationary systems close to operating points exhibit a transition to universal reconstructibility for sufficiently weak coupling, independent of the interaction topology.

## Model and methods

Consider the dynamics

$$\begin{array}{c} \tau_{\text{gl}}{\dot{x}}_{i}=-x_{i}+\alpha \sum_{j=1}^{N}A_{ij}\left(x_{j}-x_{i}\right)+\gamma \eta_{i}\left(t\right) \end{array}$$ (1)

of network dynamical systems characterized by variables **x** = (*x*_1_, …, *x*_*N*_) that interact diffusively with generic coupling strength *α* > 0 on a network topology given by an adjacency matrix *A*. The units are driven by independent white noise *η*_*i*_(*t*) of strength *γ* and relax on a time scale *τ*_gl_ > 0. The entries of the weighted adjacency matrix are *A*_*ij*_ > 0 if unit *j* physically acts on *i*, with all other elements, including the diagonal being *A*_*ij*_ = 0. Without loss of generality, we rescale time such that *τ*_gl_ = 1.

The diffusive coupling considered here emerges in approximations of coupled oscillator networks [28, 29], in population dynamics [30, 31] and in stochastic processes as, e.g., epidemic models [32].

Other types of linear coupling (e.g., in [33]) can to the same extent be treated using diffusive coupling if individual self-coupling terms are introduced (see S1 Supplementary Material). This study thus also covers networks of the form ${\dot{x}}_{i}=-c_{i}x_{i}+\alpha \sum_{j=1}^{N}A_{ij}x_{j}+\gamma_{i}\eta_{i}\left(t\right)$. For the detailed analysis of factors that hinder reconstructibility we omit individual self-coupling terms to avoid unequal scaling of correlations to establish ideal conditions for correlation thresholding.

The dynamics generated by (1) characterizes linear systems as well as stationary systems sufficiently close to given operating points.

**Fig 1:**
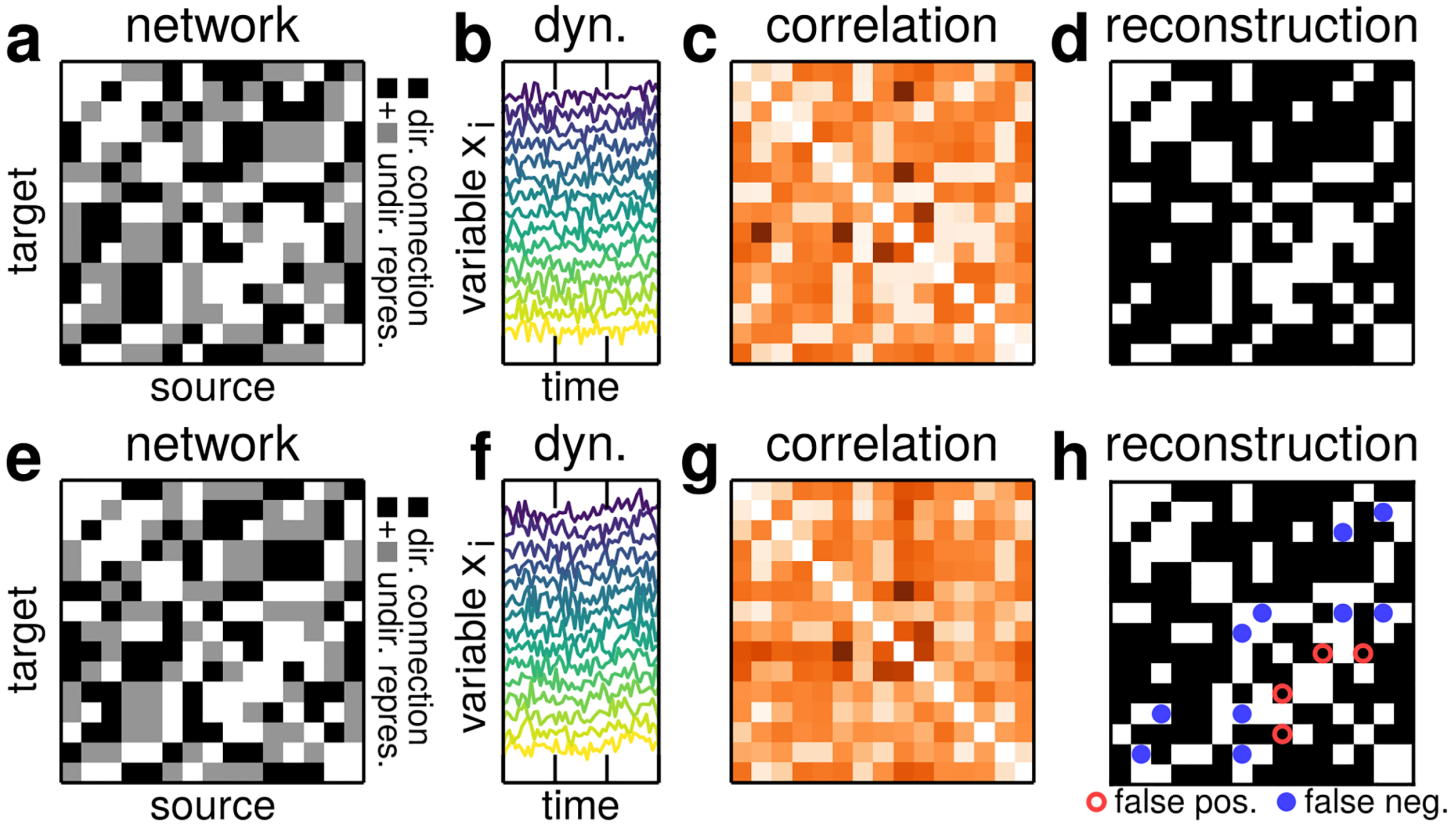
(color online) Topology-induced limits of reconstructibility. Reconstructing interaction networks from correlation thresholding may or may not yield correct connectivity pattern. (a)-(d) Successful reconstruction of a network (*N* = 15, average degree $\bar{k}=5$, *γ* = 1, *α* = 2, *A*_*ij*_ ∈ {0, 1} for absent and present interactions, resp.). (e)-(h) Reconstruction of statistically similar network is unsuccessful for any threshold. (a,e) Adjacency matrix of original network (black indicated directed interaction, gray undirected network aimed for). (b,f) Dynamics of the units yielding (c,g) correlation matrices. Thresholding yields (d) correct or (h) incorrect reconstruction, depending on the exact topology.

Can we infer the physical topology from optimally thresholding the matrix *C* of pairwise correlations (Fig 1)? The covariance matrix *σ* defined by the elements

$$\begin{array}{c} \sigma_{ij}=\langle x_{i}x_{j}\rangle -\langle x_{i}\rangle \langle x_{j}\rangle \end{array}$$ (2)

computed using an unbiased time-average 〈⋅〉, yields the correlations

$$\begin{array}{c} C_{ij}=\frac{\sigma_{ij}}{\sqrt{\sigma_{ii}\sigma_{jj}}} \end{array}$$ (3)

by normalization.

Reconstructing the physical topology implies detecting non-zero elements in the coupling matrix *A*. Also, as correlation matrices are symmetric by construction, *C*_*ij*_ = *C*_*ji*_, we relax the problem to the reconstruction of the undirected representation of the physical interaction network. Thus, we aim for the correct reconstruction of the matrix *A*′ the elements of which are given by

$$\begin{array}{c} A_{ij}^{\prime }=\{\begin{array}{cc} 1 & \text{if}\;A_{ij}=1\;\text{or}\;A_{ji}=1\\ 0 & \text{otherwise} \end{array}. \end{array}$$ (4)

Correlations (3) may be thresholded using a (possibly optimized) threshold *θ* to yield an estimate $\hat{A^{\prime }}$ with elements ${\hat{A^{\prime }}}_{ij}=1$ if *C*_*ij*_ > *θ* and ${\hat{A^{\prime }}}_{ij}=0$ otherwise. Below we focus on the question whether there is any threshold of the correlation matrix (3) that yields a correct estimate of *A*′. If there is no such threshold, we call the network non-reconstructible (in this sense).

The theory of Ornstein-Uhlenbeck processes [34] yields an analytical expression for the covariance matrix

$$\begin{array}{c} \sigma =\gamma^{2}\int_{0}^{\infty }e^{Jt}e^{J^{T}t}\;\text{d}t. \end{array}$$ (5)

Here, the matrix *J* is given by its elements

$$\begin{array}{c} J_{ij}=\{\begin{array}{cc} -(1+\alpha \sum_{j=1}^{N}A_{ij}) & \text{if}\;i=j\\ \alpha A_{ij} & \text{otherwise}. \end{array}\; \end{array}$$ (6)

**Fig 2:**
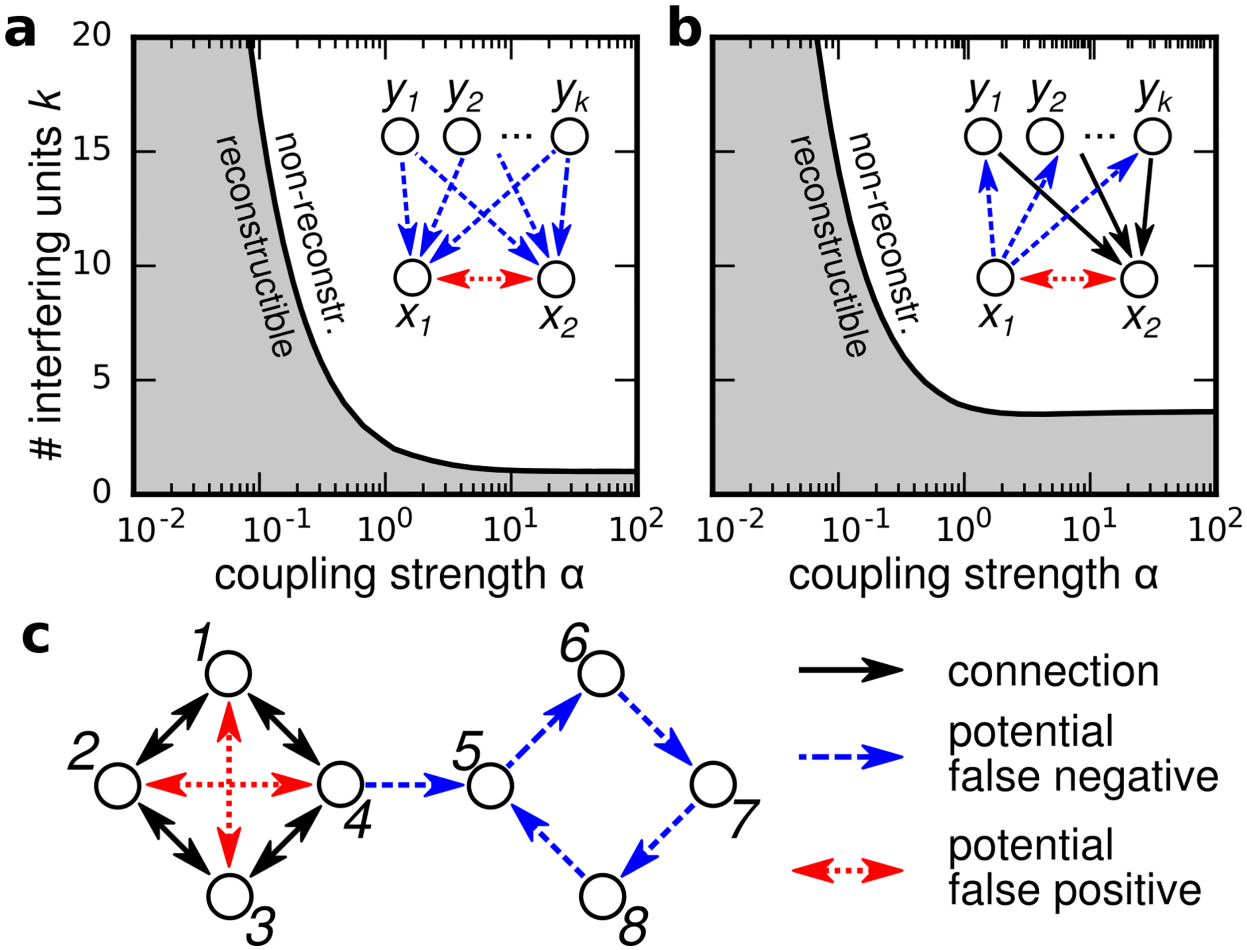
(color online) Topological sources of reconstruction errors and impact of coupling strenghts. (Unspecified parameters as in Fig 1) (a,b) Regions of reconstructible (shaded gray) and non-reconstructible networks (white shading) are non-linearly separated for (a) common cause structures and (b) relay structures (Regimes computed by interpolating analytic results using (3) and (5), details see S1 Supplementary Material). (c) Non-local effect renders larger networks non-reconstructible: Each circle would be reconstructible alone, but the joint network is not (*α* = 2).

The integral (5) can be used to compute the covariance matrix *σ* of specific network topologies with special symmetries (see Fig 2 and S1 Supplementary Material). However, numerical computation of (5) for a random network is computationally not practical.

Partial integration of (5) yields the Lyapunov equation

$$\begin{array}{c} J\sigma +\sigma J^{\text{T}}+\gamma^{2}I=0 \end{array}$$ (7)

which we solve numerically [35] to obtain the covariance matrix *σ* for explicit network topologies (*α*, *γ*, *A*). Again, (7) can be solved analytically if needed [36]. Via the relation (7) and (3), we thus semi-analytically obtain all the real-valued elements *C*_*ij*_ of the correlation matrix without any sampling error.

We order those to determine whether there is a threshold *θ* separating all existing from all non-existing links.

## Results

### Topology-induced limits of reconstructibility

Even under these idealized conditions, physical interactions are in general not reconstructible from thresholding the correlation matrix *C*. Whereas some topologies can be reconstructed via a threshold that separates existing from absent links (Fig 1a–1d), many attempted reconstructions yield false positive and false negative predictions of links, independent of the threshold (Fig 1e and 1f) and are thus intrinsically non-reconstructible by correlation thresholding.

Topologically induced errors and ultimately the limits in reconstructibility can be of local or of non-local nature (Fig 2): For instance, common input might cause unconnected units to be more correlated than connected units, a dilemma known as the common cause effect (Fig 2a inset). Likewise, two units may be strongly correlated if the network provides connectivity between them across a set of intermediate units, thereby forming a relay structure (Fig 2b inset). For both settings, reconstructibility non-linearly depends on a combination of overall coupling strength and the number of interfering units in a systematic way (Fig 2a and 2b, main panels).

In larger networks with diameter *d* ≥ 3, additional non-local effects limit reconstructibility (illustrated in Fig 2c). Differences in the correlation strength may, for instance, be caused by different link densities in different parts of the network, and imply incorrect link classification.

### Universal transition to reconstructibility

**Fig 3:**
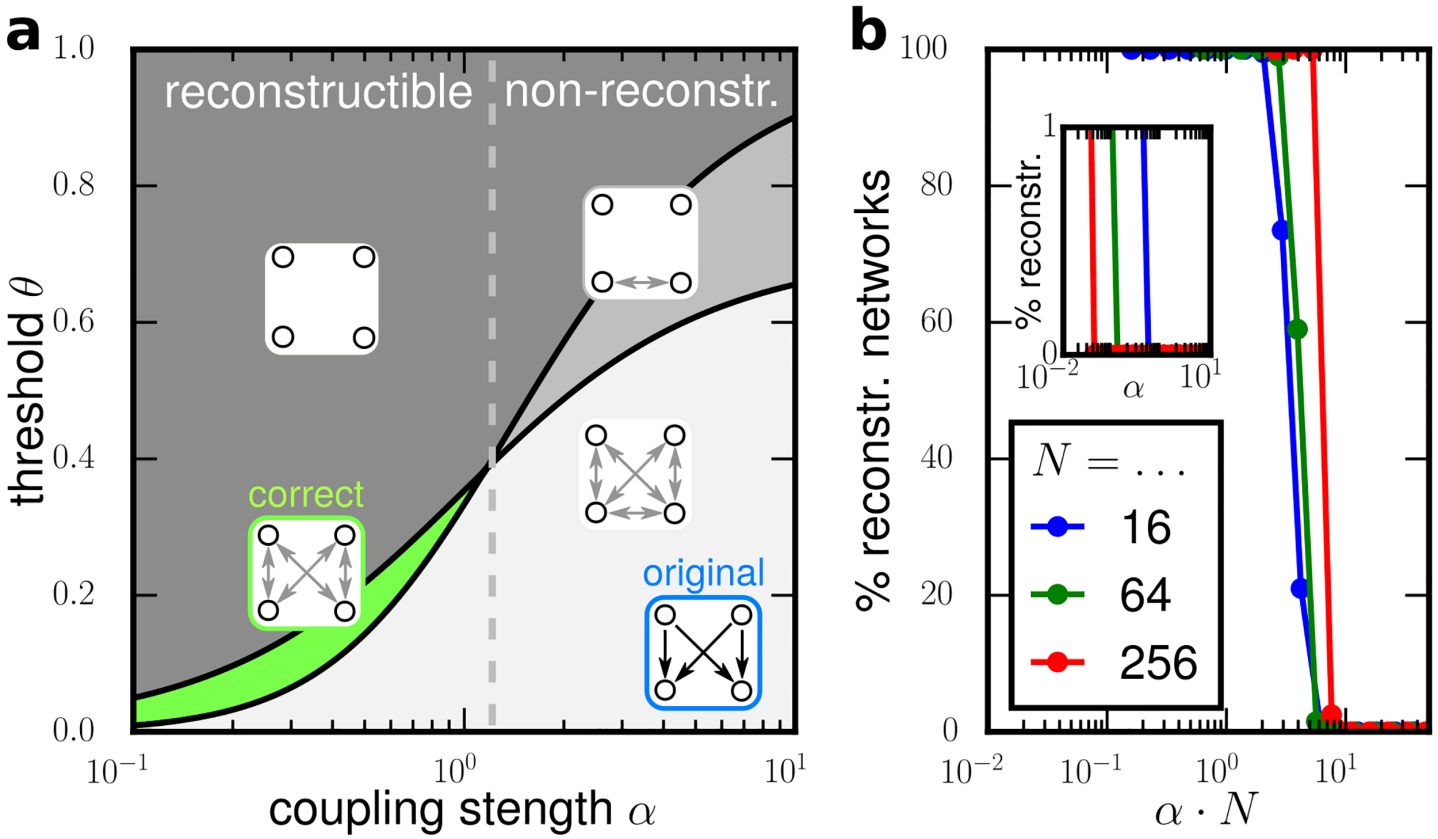
Transition to reconstructibility for weak coupling. (a) Correlation thresholding yields different estimators (shaded areas with graphs as insets) for a given topology (adjacency matrix on bottom right) depending on coupling strength and threshold. For sufficiently small coupling strength *α* (left of gray dashed line), there are ideal thresholds yielding perfect reconstruction (green shading). (Analytic results obtained using (3) and (5).) (b) Fraction of reconstructible networks exhibits transition to full reconstructibility at positive coupling strength *α* (inset) and *αN* (main panel), illustrated for random networks of *N* ∈ {16, 64, 256} units and link probability *p* = 0.5. Every arbitrary network exhibits such a transition individually (see text).

The coupling strength *α* controls the impact of both, local and non-local influences on reconstructibility. For instance, analytic treatment of a small common cause structure (Fig 3) reveals that the system becomes reconstructible for all sufficiently small coupling strengths *α* while it is non-reconstructibility if *α* is too large. This systematic transition prevails for any number of common input units in common cause structures as well as for any number of relay units in relay structures (See S1 Supplementary Material for detailed derivations).

Interestingly, all topology-induced limits disappear for sufficiently weak coupling, as seen from the following analytic argument: Rewriting the matrix

$$\begin{array}{c} J=-(1+\alpha L) \end{array}$$ (8)

in terms of the graph Laplacian *L* with elements

$$\begin{array}{c} L_{ij}=-A_{ij}+\delta_{ij}\sum_{j}A_{ij} \end{array}$$ (9)

(where *δ*_*ij*_ = 1 if *i* = *j* and zero otherwise is the Kronecker-delta) and expanding (5) for *α* ≪ 1 yields

$$\begin{array}{c} \begin{array}{cc} \sigma & =\underset{\sigma^{\left(0\right)}}{\underset{︸}{\frac{\gamma^{2}}{2}1}}-\alpha \underset{\sigma^{\left(1\right)}}{\underset{︸}{\frac{\gamma^{2}}{4}\left(L+L^{⊤}\right)}}\\ & +\alpha^{2}\underset{\sigma^{\left(2\right)}}{\underset{︸}{\frac{\gamma^{2}}{4}({LL}^{⊤}+\frac{L^{2}+L^{{⊤}^{2}}}{2})}}+O\left(\alpha^{3}\right). \end{array} \end{array}$$ (10)

The term *σ*^(1)^ on the r.h.s. of (10) does only contribute to entries *σ*_*ij*_ that reflect existing links because otherwise *L*_*ij*_ = *A*_*ij*_ = 0. Thus, the covariance of coupled units scales linearly with *α* whereas for uncoupled units it scales quadratically. So for sufficiently small coupling strength *α*, covariances of coupled units will be larger than those of uncoupled units. This result transfers to the elements of the correlation matrix *C* in (3) because diagonal elements of the covariance matrix *σ* are of order

$$\begin{array}{c} \sigma_{ii}=O\left(\alpha^{0}\right)\;\text{as}\;\alpha \;\to \;0. \end{array}$$ (11)

Hence, every network topology is reconstructible for sufficiently small coupling strengths.

Our analysis reveals, that the expansion of the covariance matrix *σ* in the coupling strength *α* is an effective separation into contributions of paths through the network with increasing lengths. The *n*th summand in the series represents the contribution of paths up to length *n*.

Similar results have been obtained in mean field models of spiking neuronal networks if the covariance matrix is self-consistently expanded in the mean neuronal input [37]. The two expansions of both approaches however differ in the order of the terms. Only expansions in network coupling strength ensure full reconstructibility of the network connectivity in the weak coupling limit.

As shown in the supplementary material, this transition to reconstructibility in the weak coupling limit is *not limited* to the considered network model and can essentially be found in all generic linear networks (for more details see S1 Supplementary Material).

### Illustrative example of reconstructibility transition

Furthermore, specific families of networks with homogeneous connectivity are reconstructible via correlation thresholding for all coupling strengths, weak and strong. As we demonstrate for illustration, this is the case for directed ring like topologies with $\bar{k}$ neighbors. In these networks the correlation matrix *C* is strictly proportional to the covariance matrix *σ* so that it is sufficient to show reconstructibility with respect to the covariance matrix. Also, since the covariance matrix *σ* is a circulant, it is sufficient to show reconstructibility only for the connections of one unit. The reconstructibility conditions is identical for all units. For simplicity of presentation, we take the number *N* of units to be even.

We order the units in such a way that it reflects the network topology, i.e.

$$\begin{array}{c} A_{i,(i+l)\mathrm{mod}N}=\{\begin{array}{cc} 1 & \text{if}\;1<l\leq \bar{k}\\ 0 & \text{otherwise} \end{array}, \end{array}$$ (12)

and replace J=-(1+αA) in (7) to obtain

$$\begin{array}{c} \sum_{l=1}^{\bar{k}}\sigma_{i,i+n-l}-2\left(\frac{1}{\alpha }+\bar{k}\right)\sigma_{i,i+n}+\sum_{l=1}^{\bar{k}}\sigma_{i,i+n+l}=-\frac{\gamma^{2}}{\alpha }\delta_{i,i+n}\; \end{array}$$ (13)

as a self-consistency equation for the entries *σ*_*ij*_ of the covariance matrix *σ*.

Here, the index *i* indicates the number of the unit in the circle and the integer *n* refers to the distance from the diagonal *n* = *j* − *i*. This nomenclature reflects the symmetry of the circulant matrix *σ*. All indices have been taken modulo the number of units *N* for simplicity.

Transforming this equation into Fourier space yields

$$\begin{array}{c} \sum_{l=1}^{\bar{k}}e^{-2\pi i\frac{lm}{N}}s_{m}-2\left(\frac{1}{\alpha }+\bar{k}\right)s_{m}+\sum_{l=1}^{\bar{k}}e^{2\pi i\frac{lm}{N}}s_{m}=-\frac{\gamma^{2}}{\alpha } \end{array}$$ (14)

with solution

$$\begin{array}{c} s_{m}=\frac{\gamma^{2}}{\alpha }\frac{1}{2\left(\frac{1}{\alpha }+k\right)-2\sum_{l=1}^{\bar{k}}\cos(2\pi \frac{lm}{N})} \end{array}$$ (15)

in Fourier coordinates. An inverse Fourier transformation yields the analytic solution

$$\begin{array}{cc} \sigma_{i,i+n-l} & =\frac{\gamma^{2}}{2+2\alpha \bar{k}+\alpha }\{\delta_{0n}+\sum_{l=1}^{\infty }\frac{\alpha^{l}\zeta_{\bar{k},n}^{*l}}{{\left(2+2\alpha \bar{k}+\alpha \right)}^{l}}\} \end{array}$$ (16)

where the sequences $\zeta_{\bar{k},n}^{*l}$ are repeated convolutions of the step sequence

$$\zeta_{\bar{k},n}=\{\begin{array}{ll} 1 & \text{if}\;n\;\text{mod}\;N\;\leq \;\bar{k}\\ 2 & \text{if}\;N\;-\;\bar{k}\;\leq \;n\;\text{mod}\;N\\ 0 & \text{otherwise} \end{array},$$ (17)

i.e.,

$$\zeta_{\bar{k}}^{*l}≔(\zeta_{\bar{k}}*\zeta_{\bar{k}}^{*(l-1)}),\;\zeta_{\bar{k}}^{*1}=\zeta_{\bar{k}}.$$ (18)

For more detailed derivations, please see the supplementary material.

Since the sequences $\zeta_{\bar{k},n}^{*}$ are monotonically decreasing in the interval *n* ∈ [−*N*/2, *N*/2] covariance only decreases with distance in the circular graph. Because for any given unit *i*, connected units are closer than non-connected units, for every such network with $\bar{k}$-regular topology, a threshold exists that separates existing from absent links, making these networks reconstructible for arbitrary coupling strengths, for any network size *N* and for any number of neighbors $\bar{k}<\frac{N}{2}$. For $\bar{k}=\frac{N}{2}$ the undirected representation of the network is fully connected and reconstruction is trivial.

### Which heterogeneities hinder reconstruction?

Given the insights from the ring-like networks, we hypothesized that if topological irregularities increase, they decrease and ultimately hinder network reconstructibility. To analyze the overall impact of topology on reconstruction quality, we investigated ensembles of directed networks in the regime between regular and random, employing a modified Watts-Strogatz small world model [38]: Starting with a regular ring of *N* units with each unit receiving directed links from $\bar{k}$ preceding nodes (and thus a mean in- and out-degree of $\bar{k}$) the source and the target of each link are detached with probability *q*_out_ and probability *q*_in_ respectively. The resulting loose ends are randomly redistributed in the network while avoiding self-loops and multiple links. This creates networks of mean degree $\bar{k}$ whose in-degree distribution $p_{k}^{\text{in}}$ and out-degree distribution $p_{k}^{\text{out}}$ are altered separately from their original values $p_{k}^{\text{in}}=p_{k}^{\text{out}}=\delta_{k\bar{k}}$ by varying *q*_in_ and *q*_out_. This random graph ensemble contains networks with unimodal degree distributions (binomial for *q*_in_ = *q*_out_ = 1, $\bar{k}⪡N$ and 1 ≪ *N*) so that the variances of the distributions serve as indicators for the inhomogeneities in the network.

**Fig 4:**
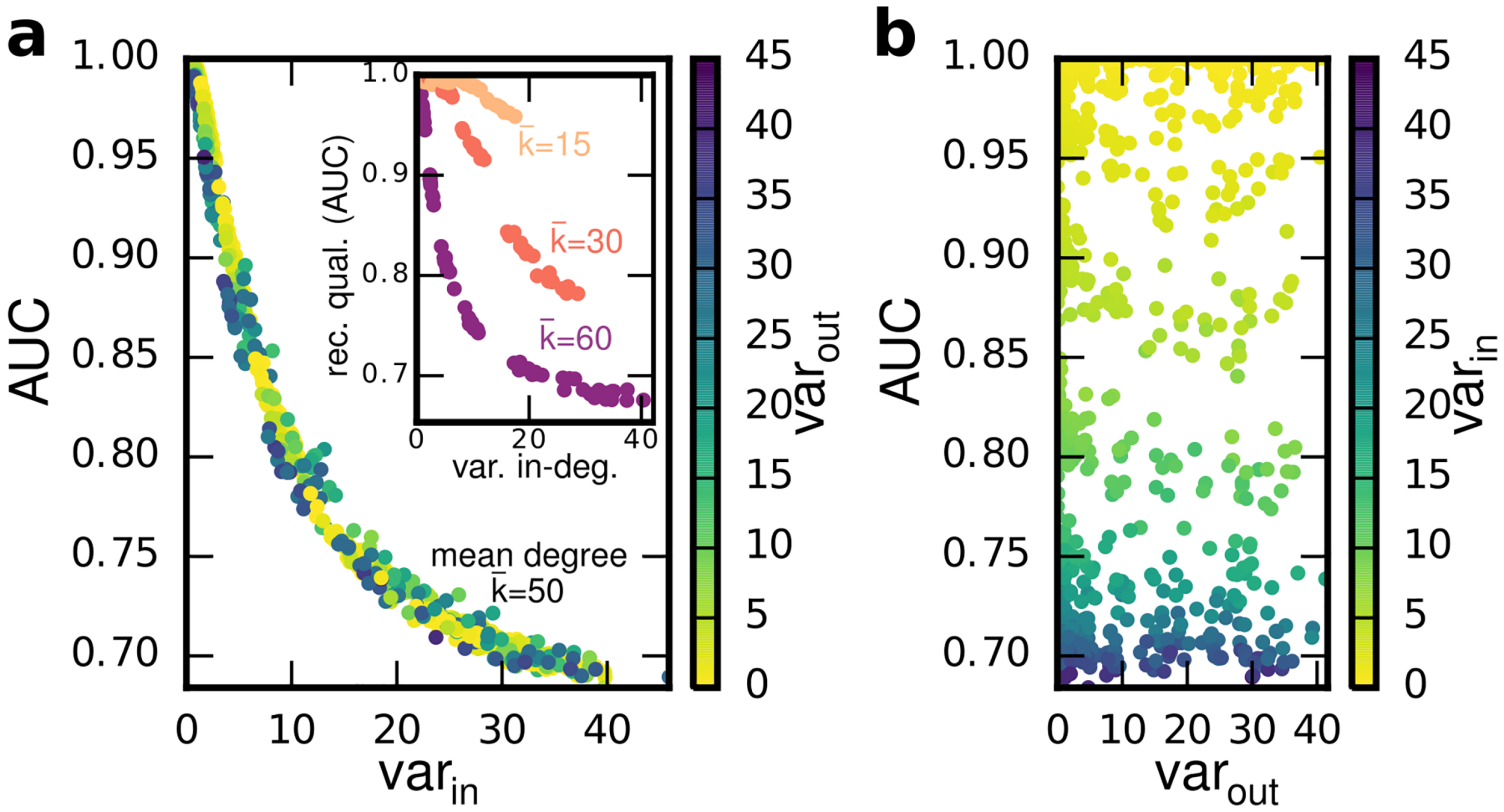
(color online) Reconstruction systematically varies with heterogeneities in in-degree, but not in out-degree. (a) AUC exhibits functional dependency on the variance of the in-degree distribution var_in_, regardless of the variance of the out-degree var_out_. Inset: Qualitative behavior is the same for differnet mean degrees. (b) No significant dependency of reconstruction quality on out-degree heterogeneity (network size *N* = 150 throughout, *α* = 1, *A*_*ij*_ ∈ {0, 1}).

Considering a fixed coupling strength (e.g., *α* = 1), we quantify reconstructibility by measuring the AUC, the area under the ROC (receiver operating characteristic) curve, generated by a variable correlation threshold *θ*. AUC ranges from AUC = 0.5 for random guessing to AUC = 1 for perfect reconstructibility (see S1 Supplementary Material for an introduction to ROC curves). For networks that are not densely connected ($\bar{k}<\left(N-1\right)/2$), we find that reconstruction quality systematically decreases with in-degree heterogeneity, with the AUC exhibiting a functional dependency on the variance of the in-degree distribution, yet is almost independent of the variance of the out-degree distribution (compare Fig 4a with Fig 4b). Thus, the reconstruction error is mainly explained by the in-degree heterogeneity. We obtain qualitatively similar results across different average connectivities $\bar{k}$ (inset of Fig 4a).

This finding coincides with intuition: Since the influence of a source unit on its target decreases with the number of additional signals its target receives (see common cause structure, S1 Supplementary Material), large differences in the in-degree directly correspond to high variability in pairwise correlations. Correlations in complex networks with inhomogeneous in-degree thus strongly depend on the local link density and can therefore not be faithfully reconstructed using a global threshold. In contrast, the number of outgoing connections does not directly impact pairwise correlations.

## Conclusions

In summary, we have systematically investigated reconstructibility of physical interaction networks from thresholding statistical correlations. Beyond valuable previous studies which targeted the impact of correlated noise and estimation errors [39, 40], we revealed intrinsic limits of reconstructibility induced by the strengths of network interactions and their topology. In particular, a number of distinct topological factors contribute in a systematic way: local common cause structures, local relay structures, in-degree heterogeneities as well as non-local structural elements of a network resulting from different link densities in different network parts. Intriguingly, for stationary dynamics and arbitrary network topologies we uncovered a transition to full reconstructibility when decreasing the coupling strengths. Whereas the exact critical coupling strength to transition to reconstructibility depends on the topology, it is guaranteed to occur for all topologies.

Given the limitations of correlation thresholding, alternate methods of reconstruction from time series data are required (e.g, [6, 7, 24, 41]). In systems of linearly coupled spiking neurons, coupling strengths may for example be reconstructed using sparse reconstruction of connections [42] if connections are sparse or covariance inversion [36, 41] if temporal information is available.

For systems that are strongly non-linear and non-stationary, the range of inference methods is currently largely limited to systems with models known a priori. Such non-linear systems in general pose a number of additional challenges, including that there typically is no well-defined, temporally constant coupling strength between the units. Future studies would need to investigate model-independent methods to obtain physical interaction structure from recorded non-linear dynamics [4–11, 24].

Our main result on full reconstructiblity in the weak coupling limit might provide a useful initial step towards the reconstruction of non-linear and non-stationary networks: By systematically combining localized but faithful reconstructions obtained from an entire set of dynamics around different operation points in weakly coupled networks a global picture of the underlying interactions and their network state-dependencies could be obtained. Our finding, that the transition to reconstructibility is observed in all generic linear networks (see S1 Supplementary Material) yields promising perspectives for future investigations.

Our results on topology-induced limits of network reconstructibilty not only further our theoretical insights about the relations between statistical correlation and physical interaction networks [23, 24, 43] but also indicate where principal care has to be taken in applications when analyzing statistical correlation data to reveal aspects of direct physical interactions.

## Supporting information

S1 Supplementary Material (PDF)
