# Supplementary material for: Transition to reconstructibility in weakly coupled networks
Source: PLoS One. 2017 Oct 20;12(10):e0186624. doi: 10.1371/journal.pone.0186624 (PMC5650155; doi:10.1371/journal.pone.0186624)
Supplement: S1 Supplementary Material — (PDF) [file pone.0186624.s001.pdf]

Supplementary Material accompanying the manuscript

Transition to reconstructibility in weakly coupled networks

Benedict J. Lünsmann,[1,2] Christoph Kirst,[1,3] and Marc Timme[1,4,5,6,7]

[1] *Network Dynamics, Max Planck Institute for Dynamics and Self-Organization (MPIDS), 37077 Göttingen, Germany*
[2] *Max Planck Institute for the Physics of Complex Systems (MPIPKS), 01187 Dresden, Germany*
[3] *Rockefeller University, NY 10065-6399 New York, USA*
[4] *Bernstein Center for Computational Neuroscience (BCCN), 37077 Göttingen, Germany*
[5] *Institute for Nonlinear Dynamics, University of Göttingen, 37077 Göttingen, Germany*
[6] *Chair for Network Dynamics, Center for Advancing Electronics Dresden (cfaed) and Institute for Theoretical Physics, Technical University of Dresden, 01062 Dresden, Germany*
[7] *Department of Physics, Technical University of Darmstadt, 64289 Darmstadt, Germany*

In parts of the main manuscript, we investigate the performance of correlation thresholding as a means of network reconstruction from time series data. In order to find the upper bound of reconstruction performance, we considered optimal conditions such as a linear model, globally constant coupling strengths and noise strengths, unique and uncorrelated white noise sources for each unit of the network and infinite time series length. We found that under these conditions weakly coupled networks as well as regular directed ring topologies are always reconstructible and that reconstruction quality depends mainly on the homogeneity of in-degrees. Mathematical proofs, detailed calculations and an introduction to ROC curves can be found in this document.

MODEL

Here, we consider networks of N units each described by a state variable x_i, $i \in \{1, \ldots, N\}$, that evolve according to an Ornstein-Uhlenbeck (OU) process given by

$$\dot{x_i} = -x_i + \alpha \sum_{j=1}^{N} A_{ij}(x_j - x_i) + \gamma \eta_i(t) \quad (1)$$

with $\dot{\boldsymbol{x}}, \boldsymbol{x} \in \mathbb{R}^N$, white noise vector $\boldsymbol{\eta}(t) \in \mathbb{R}^N$, adjacency matrix $A_{ij} \in \{0,1\}^{N\times N}$, coupling strength $\alpha \in \mathbb{R}_+$ and noise strength $\gamma \in \mathbb{R}_+$.

Introducing the Laplace matrix L with elements

$$L_{ij} = -A_{ij} + \delta_{ij} \sum_{k=1}^{N} A_{ik} \quad (2)$$

(where δ_{ij} is the Kronecker-delta) and the drift matrix

$$J = -(\mathbb{1} + \alpha L) \quad (3)$$

the process (1) can be rewritten in the multivariate form

$$\dot{\boldsymbol{x}} = J\boldsymbol{x} + \gamma \boldsymbol{\eta}(t). \quad (4)$$

Since the drift matrix J is diagonally negative dominant, it has only eigenvalues with non-zero negative real part, so that the process has a stationary solution with covariance matrix

$$\sigma = \gamma^2 \int_0^\infty e^{Jt} e^{J^\intercal t} \, \mathrm{d}t \quad (5)$$

that fulfills the Lyapunov equation

$$J\sigma + \sigma J^\intercal + \gamma^2 \mathbb{1} = 0 \; . \quad (6)$$

For reference see [1].

The existence of an analytic equation for the covariance matrix σ enables us to compute the covariance matrix directly without simulating the process, avoiding additional errors induced by finite time series.

DETAILED ANALYTIC DERIVATION OF CORRELATIONS

Here, we present the detailed analytic derivation of the analytic correlations in the generalized common cause problem and the generalized relay structure problem.

We proceed as follows:
First, we compute the instantaneous covariance matrix σ of the OU process by solving the integral given by (5), or more precisely

$$\sigma = \frac{\gamma^2}{\alpha} \int_0^\infty e^{-\frac{2}{\alpha}t'} \underbrace{e^{-Lt'} e^{-L^\intercal t'}}_{=:\Lambda(t')} \, \mathrm{d}t' \, . \tag{7}$$

For this purpose, we calculate the matrix $\Lambda(t)$, which is determined by the topology, and integrate element-wise to get elements of the matrix σ.
Then, we compute the Pearson correlation matrix C using its definition $C_{ij} = \frac{\sigma_{ij}}{\sqrt{\sigma_{ii}\sigma_{jj}}}$.

For Fig. 2a,b in the manuscript, we then calculate the difference in correlation for existing connections and non-existing connections as a function of coupling strength α and number of source units (common cause problem) m or transmitting units (relay structure) m and interpolate the zero-crossing of this difference in α-m space numerically.

Common Cause Structure

Let $\boldsymbol{Y} = (Y_1, Y_2, \ldots, Y_m) \in \mathbb{R}^m$, $\boldsymbol{X} = (X_1, X_2) \in \mathbb{R}^2$ be two vectors of unit representing random variables and let each element of $\boldsymbol{Y}$ be a source unit of each element of $\boldsymbol{X}$. Then, the topology A and the Laplacian L for the network of the process $\boldsymbol{Z} = (\boldsymbol{X}, \boldsymbol{Y})$ are given by

$$A = \begin{pmatrix} 0 & 0 & 1 & 1 & \cdots \\ 0 & 0 & 1 & 1 & \cdots \\ 0 & 0 & 0 & 0 & \cdots \\ \vdots & \vdots & \vdots & \vdots & \ddots \end{pmatrix} \quad \Rightarrow \quad L = \begin{pmatrix} m & 0 & -1 & -1 & \cdots \\ 0 & m & -1 & -1 & \cdots \\ 0 & 0 & 0 & 0 & \cdots \\ \vdots & \vdots & \vdots & \vdots & \ddots \end{pmatrix} \, . \tag{8}$$

The matrix power of L yields

$$L^n = \begin{cases} m^{n-1} L & n \neq 0 \\ \mathbb{1} & n = 0 \end{cases} \quad n \in \mathbb{N} \, . \tag{9}$$

Thus, the matrix exponential is given by

$$\begin{aligned} e^{-Lt} &= \sum_{n=0}^{\infty} \frac{(-t)^n}{n!} L^n \\ &= \mathbb{1} + \sum_{n=1}^{\infty} \frac{(-t)^n m^{n-1}}{n!} L \\ &= \mathbb{1} + \frac{e^{-mt} - 1}{m} L \, . \end{aligned} \tag{10}$$

Hence,

$$\Lambda(t) := e^{-Lt} e^{-L^\intercal t} = \mathbb{1} + \frac{e^{-mt} - 1}{m}(L + L^\intercal) + \left(\frac{e^{-mt} - 1}{m}\right)^2 L L^\intercal \tag{11}$$

with

$$LL^T = \begin{pmatrix} m^2 + m & m & 0 & \cdots \\ m & m^2 + m & 0 & \cdots \\ 0 & 0 & 0 & \cdots \\ \vdots & \vdots & \vdots & \ddots \end{pmatrix} \, , \tag{12}$$

so that the entries of Λ are given by

$$\Lambda_{11} = \Lambda_{22} = 1 + 2(e^{-mt} - 1) + \frac{m^2 + m}{m^2}(e^{-mt} - 1)^2 \tag{13}$$

$$\Lambda_{33} = \ldots = \Lambda_{NN} = 1 \tag{14}$$

$$\Lambda_{12} = \frac{(e^{-mt} - 1)^2}{m} \tag{15}$$

$$\Lambda_{13} = \ldots = \Lambda_{1N} = \Lambda_{23} = \ldots = \Lambda_{2N} = \Lambda_{13} = -\frac{e^{-mt} - 1}{m} \ . \tag{16}$$

All remaining entries not defined by $\Lambda = \Lambda^\intercal$ are zero.
Integrating

$$\sigma_{ij} = \frac{\gamma^2}{\alpha} \int_0^\infty e^{-\frac{2}{\alpha}t} \Lambda_{ij}(t) \, \mathrm{d}t \tag{17}$$

yields

$$\sigma_{11} = \sigma_{22} = \gamma^2 \frac{\alpha^2 m + \alpha m + 2}{(\alpha 2 + 2)(2\alpha m + 2)} \tag{18}$$

$$\sigma_{33} = \ldots = \sigma_{NN} = \frac{\gamma^2}{2} \tag{19}$$

$$\sigma_{12} = \gamma^2 \frac{\alpha^2 m}{(\alpha m + 2)(2\alpha m + 2)} \tag{20}$$

$$\sigma_{13} = \ldots = \sigma_{1N} = \sigma_{23} = \ldots = \sigma_{2N} = \sigma_{13} = \frac{\gamma^2}{2} \frac{\alpha m}{\alpha m + 2} \ . \tag{21}$$

Normalizing yields two different correlation values: The correlation

$$C_{xx} = \frac{\alpha^2 m}{\alpha^2 m + \alpha m + 2} \tag{22}$$

of the non-connected nodes X_1 and X_2 and the correlation

$$C_{xy} = \sqrt{\left(\frac{\alpha m + 2}{\alpha m + 4}\right)\left(\frac{\alpha^2}{\alpha^2 m + \alpha m + 2}\right)} \tag{23}$$

for connection from units in $\boldsymbol{Y}$ to units in $\boldsymbol{X}$.

For Fig. 2a of the main article, we determined the difference between correlations of unconnected pairs and connected pairs $C_{xx} - C_{xy}$ in dependence on the coupling strength α and the number of source units m and plotted the zero crossing in α-m space. This curve marks the transition from reconstructible to non-reconstructible.

Relay Structures

We perform the same analysis that was done for the common cause structure (see above) for the relay structure.

Here, we define $\boldsymbol{Z} = (X_2, \boldsymbol{Y}, X_1)^\intercal$. Each element of $\boldsymbol{Y}$ gets inputs from X_1 and each element of $\boldsymbol{Y}$ is a source unit of X_2.
The adjacency matrix and the Laplacian of the network for $\boldsymbol{Z}$ are

$$A = \underbrace{\begin{pmatrix} 0 & 1 & \cdots & 1 & 0 \\ 0 & 0 & \cdots & 0 & 1 \\ \vdots & \vdots & \ddots & \vdots & \vdots \\ 0 & 0 & \cdots & 0 & 1 \\ 0 & 0 & \cdots & 0 & 0 \end{pmatrix}}_{m+2} \qquad \Rightarrow \qquad L = \begin{pmatrix} m & -1 & \cdots & -1 & 0 \\ 0 & 1 & \cdots & 0 & -1 \\ \vdots & \vdots & \ddots & \vdots & \vdots \\ 0 & 0 & \cdots & 1 & -1 \\ 0 & 0 & \cdots & 0 & 0 \end{pmatrix} \ . \tag{24}$$

The matrix power of the Laplacian yields

$$L^n = \begin{pmatrix} m^n & -\frac{1-m^n}{1-m} & \cdots & -\frac{1-m^n}{1-m} & \frac{m-m^n}{1-m} \\ 0 & 1 & \cdots & 0 & -1 \\ \vdots & \vdots & \ddots & \vdots & \vdots \\ 0 & 0 & \cdots & 1 & -1 \\ 0 & 0 & \cdots & 0 & 0 \end{pmatrix}, \tag{25}$$

where used the geometric series.
Hence, the matrix exponential is given by

$$e^{-Lt} = \begin{pmatrix} e^{-mt} & \frac{e^{-t}-e^{-mt}}{m-1} & \cdots & \frac{e^{-t}-e^{-mt}}{m-1} & \frac{m(1-e^{-t})-1+e^{-mt}}{m-1} \\ 0 & e^{-t} & \cdots & 0 & 1-e^{-t} \\ \vdots & \vdots & \ddots & \vdots & \vdots \\ 0 & 0 & \cdots & e^{-t} & 1-e^{-t} \\ 0 & 0 & \cdots & 0 & 1 \end{pmatrix}. \tag{26}$$

The matrix Λ and the covariance matrix σ are computed following the same ideas as in the previous paragraph. We find four correlation values: Two for the existing connections $X_1 \to Y_i$

$$C_{xy} = \sqrt{\left(\frac{1+\alpha}{2+\alpha}\right)\left(\frac{\alpha^2}{\alpha^2+\alpha+2}\right)} \tag{27}$$

and $Y_i \to X_1$

$$\begin{aligned} C_{yx} = {} & \alpha\left(\alpha^3(m^2+m)+4\alpha^2m+2\alpha(m+1)+4\right)\sqrt{\alpha m+1}\cdot \\ & \frac{\sqrt{(\alpha m+2)(\alpha^2+\alpha+2)(\alpha m+\alpha+2)(\alpha^5(m^3+m^2)+}}{\alpha^4m(5m+1)+\alpha^3(5m^2+9m+2)+2\alpha^2(m^2+9m+5)+8\alpha(m+2)+8)}^{-1} \end{aligned} \tag{28}$$

and two for the non-existing connections $Y_i \leftrightarrow Y_j$

$$C_{yy} = \frac{\alpha^2}{\alpha^2+\alpha+2} \tag{29}$$

and $X_1 \leftrightarrow X_2$

$$\begin{aligned} C_{xx} = {} & \alpha^2 m\sqrt{(\alpha+1)(\alpha m+1)(\alpha m+\alpha+2)(\alpha+2)(\alpha m+2)}\sqrt{\alpha^5m^2(m+1)+} \\ & \overline{\alpha^4m(5m+1)+\alpha^3(5m^2+9m+2)+2\alpha^2(m^2+9m+5)+8\alpha(m+2)+8}^{-1} \end{aligned} \tag{30}$$

As for common cause structures, we compute the difference between the correlation of unconnected units C_{xx} and the smallest correlation among connected units C_{xy} and determine the zero-crossing in α-m space. Like before, this curve marks the transition from reconstructible to non-reconstructible.

RECONSTRUCTIBILITY IN THE WEAK COUPLING LIMIT

Specific Network Model

Resolving $J = -(\mathbb{1} + \alpha L)$ in (5) yields

$$\sigma = \gamma^2 \int_0^\infty e^{-2t} e^{-\alpha L t} e^{-\alpha L^\intercal t} \, \mathrm{d}t \, . \tag{31}$$

Since the matrix exponential is defined as

$$e^{-\alpha L t} := \sum_{n=0}^\infty \frac{\alpha^n t^n}{n!} L^n \tag{32}$$

$$= \mathbb{1} + \alpha t L + \frac{\alpha^2 t^2}{2} L^2 + \mathcal{O}(\alpha^3) \tag{33}$$

with finite rest $\mathcal{O}(\alpha^3)$, the integral can be written as

$$\sigma = \gamma^2 \int_0^\infty \exp(-2t) \left(\mathbb{1} - \alpha L t + \frac{\alpha^2 t^2}{2} L^2 + \dots \right) \left(\mathbb{1} - \alpha L^\intercal t + \frac{\alpha^2 t^2}{2} L^{\intercal 2} + \dots \right) \, \mathrm{d}t \tag{34}$$

$$= \gamma^2 \int_0^\infty \exp(-2t) \Big(\mathbb{1} - \alpha (L + L^\intercal) t + \frac{\alpha^2 t^2}{2} (2 L L^\intercal + L^2 + L^{\intercal 2}) \Big) + \mathcal{O}(\alpha^3) \, \mathrm{d}t \tag{35}$$

$$= \gamma^2 \left\{ \frac{1}{2} \mathbb{1} - \frac{\alpha}{4} (L + L^\intercal) + \frac{\alpha^2}{8} (2 L L^\intercal + L^2 + L^{\intercal\, 2}) + \mathcal{O}(\alpha^3) \right\} . \tag{36}$$

Hence, diagonal elements of the covariance matrix σ are given by

$$\sigma_{ii} = \frac{\gamma^2}{2} + \mathcal{O}(\alpha^1) \, , \tag{37}$$

elements corresponding to links are given by

$$\sigma_{ij}^{\mathrm{c}} = -\frac{\gamma^2 \alpha}{4} (L_{ij} + L_{ji}) + \mathcal{O}(\alpha^2) \, , \tag{38}$$

and elements corresponding to non-links are given by

$$\sigma_{kl}^{\mathrm{nc}} = \frac{\gamma^2 \alpha^2}{8} \overbrace{(2 L L^\intercal + L^2 + L^{\intercal\, 2})_{kl}}^{M_{kl}} + \mathcal{O}(\alpha^3) \, . \tag{39}$$

Hence, elements of the correlation matrix C belonging to connections are given by

$$C_{ij}^{\mathrm{c}} = -\frac{1}{2} \frac{\alpha (L_{ij} + L_{ji}) + \mathcal{O}(\alpha^2)}{1 + \mathcal{O}(\alpha^1)} \tag{40}$$

and elements of the correlation matrix C corresponding to non-connections are given by

$$C_{kl}^{\mathrm{nc}} = \frac{1}{4} \frac{\alpha^2 M_{kl} + \mathcal{O}(\alpha^3)}{1 + \mathcal{O}(\alpha^1)} \, . \tag{41}$$

For weak coupling strength $\alpha \ll 1$ this ensures that there is a critical coupling strength $\alpha_c(A)$ for which every coupling strength $\alpha < \alpha_c(A)$ results in $C_{ij}^{\mathrm{c}} > C_{kl}^{\mathrm{nc}}$ for all indices i, j, k, l. Hence, there exists a threshold $\theta(\alpha, A)$ for the correlation matrix C that results in the reconstruction of the original network A.

General Network Model

Reconstructibility in the weak coupling limit persists even the more general network given by the stochastic differential equation

$$\dot{x}_i = -c_i x_i + \alpha \sum_{j=1}^{N} A_{ij} x_j + \gamma_i \eta_i(t) \tag{42}$$

where $c_i > 0$ is the self-coupling term, $\alpha > 0$ is the coupling parameter, $A \in \mathbb{R}_+^{N \times N}$ is the coupling matrix with diagonal elements $A_{ii} = 0$ and γ_i is the noise strength of unit i.

Eq. (42) can be written as the matrix equation

$$\dot{\boldsymbol{x}} = \underbrace{(-D_c + \alpha A)}_{M} \boldsymbol{x} + D_\gamma \boldsymbol{\eta}(t) \tag{43}$$

with diagonal matrices D_c and D_γ as $D_y = \mathrm{diag}(y_1, \ldots, y_N)$.

We demand the matrix M to have eigenvalues with non-vanishing negative real part for this Ornstein-Uhlenbeck process to be stable. Otherwise a covariance matrix is not defined.

The covariance matrix σ of the process described by Eq. (43) fulfills the Lyapunov equation

$$M\sigma + \sigma M^\intercal + D_\gamma^2 = 0 \tag{44}$$

$$\Rightarrow D_c \sigma + \sigma D_c = \alpha(A\sigma + \sigma A^\intercal) + D_\gamma^2 \ . \tag{45}$$

Using a perturbation ansatz in the coupling term α for the covariance matrix σ

$$\sigma = \sigma^{(0)} + \alpha\sigma^{(1)} + \alpha^2\sigma^{(2)} + \ldots \tag{46}$$

yields

$$D_c \sigma^{(0)} + \sigma^{(0)} D_c = D_\gamma^2 \ . \tag{47}$$

$$\Rightarrow \sigma^{(0)} = \frac{1}{2} D_\gamma^2 D_c^{-1} \tag{48}$$

and

$$D_c \sigma^{(n+1)} + \sigma^{(n+1)} D_c = A\sigma^{(n)} + \sigma^{(n)} A^\intercal \tag{49}$$

$$\Rightarrow \sigma_{ij}^{(n+1)} = \frac{1}{c_i + c_j} \left[A\sigma^{(n)} + \sigma^{(n)} A^\intercal \right]_{ij} \ . \tag{50}$$

Hence, the first order term $\sigma^{(1)}$ is given by

$$\Rightarrow \sigma_{ij}^{(1)} = \frac{1}{2(c_i + c_j)} \left(A_{ij} \frac{\gamma_j^2}{c_j} + A_{ji} \frac{\gamma_i^2}{c_i} \right) \ . \tag{51}$$

Its elements $\sigma_{ij}^{(1)}$ are zero if and only if A_{ij} and A_{ji} are zero. This means that the second order term

$$\sigma_{ij}^{(2)} = \frac{1}{c_i + c_j} \left(\sum_{k=1}^{N} A_{ik} \sigma_{kj}^{(1)} + \sigma_{ik}^{(1)} A_{jk} \right)_{ij} \tag{52}$$

is only non-zero if either $\left[A^2\right]_{ij}$, $[AA^\intercal]_{ij}$ or $\left[A^{2\intercal}\right]_{ij}$ are zero.

Hence, the second order term $\sigma^{(2)}$ is generated by second order connections and is an effective perturbation to the first order connections which are reflected by the first order term $\sigma^{(1)}$.

The network topology is reconstructible by correlation thresholding if there exists a correlation threshold θ that separates absolute correlation values of non-connections (i.e., pairs of units (i, j) for which $A_{ij} = 0$) and absolute correlation values of connections (i.e., pairs of units (i, j) for which $A_{ij} \neq 0$).

Since the covariance matrix σ is given by

$$\sigma \approx \sigma^{(0)} + \alpha\sigma^{(1)} + \alpha^2\sigma^{(2)} \ , \tag{53}$$

the perturbation term $\sigma^{(2)}$ decreases faster than the coupling term $\sigma^{(1)}$ for small coupling strengths α. Hence, every network must become reconstructible in the weak coupling limit.

The same argument can in general not be made for $A \in \mathbb{R}^{N\times N}$ since elements in the first order term $\sigma^{(1)}$ might vanish.

However, these networks are finely tuned and represent a zero set in the space of all coupling topologies. Thus, all generic linear networks become reconstructible in the weak coupling limit $\alpha \ll 1$.

RECONSTRUCTIBILITY OF CIRCLES

We proof that any directed circular topology results in a correlation matrix C that can be thresholded such that the original network topology A is retrieved. Hence, any circular topology is reconstructible by correlation thresholding.

The proof goes as follows:

1. We demonstrate that the correlation between units decreases monotonically with distance in the circle.

2. We show that every unit is more correlated with its farthermost connected unit than with its closest unconnected unit.

3. We conclude that every pair of connected units is stronger correlated than any pair of non-connected units such that the network is reconstructible by correlation thresholding.

Proof of Monotonicity

From (6) we obtain

$$\sigma_{ij} = \frac{1}{2+\alpha(k_{\mathrm{in},i}+k_{\mathrm{in},j})}\left(\gamma^2\delta_{ij} + \alpha\left[\sum_{\{l:i\leftarrow l\}}\sigma_{jl} + \sum_{\{l:j\leftarrow l\}}\sigma_{li}\right]\right) \ , \tag{54}$$

as a relation between elements of the covariance matrix σ. Here, δ_{ij} is the Kronecker-delta, $k_{\mathrm{in},i}$ ist the in-degree of unit i and $\sum_{\{l:i\leftarrow l\}}$ is the sum over all indices of units that are in-neighbors of unit i.

The topology of the network determines how to resolve the two sums. In case of directed k-rings each units gets input from the subsequent k units. In addition, the in-degree for each node is k. Hence,

$$\sigma_{ij} = \frac{1}{2+2\alpha k}\left(\gamma^2\delta_{ij} + \alpha\left[\sum_{l=1}^{k}\sigma_{j,i+l} + \sum_{l=1}^{k}\sigma_{j+l,i}\right]\right) \ . \tag{55}$$

In a k-ring k is the maximum distance between connected units, for this reason $2k+1 < N$. Equality denotes a network in which all units are already connected either by incomming or outgoing connections, so that a reconstruction is trivial because no unconnected pairs exist.

The topological features of a k-ring have further consequences: Due to the fact that such a graph is rotationally invariant, the covariance between two units only depends on the distance in the ring. Thus, σ is a circulant matrix, i.e. $\sigma_{(i+n) \mod N,(j+n) \mod N} = \sigma_{ij}$ for all $n \in \mathbb{Z}$. This means, σ is fully determined by the sequence $(\sigma_{i,i+n})_{n=0}^{N-1}$. Also, the correlation values $C_{ij} := \frac{\sigma_{ij}}{\sqrt{\sigma_{ii}\sigma_{jj}}} = \frac{\sigma_{i,i+n}}{\sigma_{ii}}$ are just proportional to the covariance values. Hence, *thresholding covariance is fully equivalent to thresholding correlation.*

For convenience, we define the periodic sequence $\varkappa \hat{=} (\varkappa_n)_{n=-\infty}^{\infty}$ with period N and $\varkappa_n := \sigma_{i,i+n}$. This sequence fulfills $\varkappa_{n+N} = \varkappa_n$ due to periodic boundary conditions for the indices. In addition, the covariance matrix σ is symmetric, i.e. $\sigma_{ij} = \sigma_{ji}$, so that the periodic sequence $\varkappa$ also has to fulfill $\varkappa_n = \varkappa_{-n}$ for all $n \in \mathbb{Z}$.

Using both symmetries (55) yields

$$\sum_{l=1}^{k}\sigma_{i,i+n-l} - 2(\frac{1}{\alpha}+k)\sigma_{i,i+n} + \sum_{l=1}^{k}\sigma_{i,i+n+l} = -\frac{\gamma^2}{\alpha}\delta_{i,i+n} \tag{56}$$

$$\Rightarrow \sum_{l=1}^{k}\varkappa_{n-l} - 2(\frac{1}{\alpha}+k)\varkappa_{n} + \sum_{l=1}^{k}\varkappa_{n+l} = -\frac{\gamma}{\alpha}\delta_{0m} \tag{57}$$

We make use of the periodicity of $\varkappa$ by applying the Fourier transform $s := \mathcal{F}[\,\varkappa\,]$.
Multiplying (57) by $e^{-2\pi i \frac{nm}{N}}$ and summing the resulting equation over all $m \in [0, N-1]$ yields

$$\sum_{m=0}^{N-1}\left\{\sum_{l=1}^{k}\varkappa_{n-l}e^{-2\pi i\frac{nm}{N}} - 2(\frac{1}{\alpha}+k)\varkappa_n e^{-2\pi i\frac{nm}{N}} + \sum_{l=1}^{k}\varkappa_{n+l}e^{-2\pi i\frac{nm}{N}}\right\} = -\frac{\gamma^2}{\alpha} \tag{58}$$

$$\Rightarrow \quad \sum_{l=1}^{k}\left\{\sum_{m=0}^{N-1}\varkappa_{n-l}e^{-2\pi i\frac{nm}{N}} - \frac{2(\frac{1}{\alpha}+k)}{k}\sum_{m=0}^{N-1}\varkappa_n e^{-2\pi i\frac{nm}{M}} + \sum_{m=0}^{N-1}\varkappa_{n+l}e^{-2\pi i\frac{nm}{N}}\right\} = -\frac{\gamma^2}{\alpha} \tag{59}$$

$$\Rightarrow \quad \sum_{l=1}^{k}e^{-2\pi i\frac{lm}{N}}s_m - 2(\frac{1}{\alpha}+k)s_m + \sum_{l=1}^{k}e^{2\pi i\frac{lm}{N}}s_m = -\frac{\gamma^2}{\alpha} \tag{60}$$

$$\Rightarrow \quad s_m = \frac{\gamma^2}{\alpha}\frac{1}{2(\frac{1}{\alpha}+k) - 2\sum\limits_{l=1}^{k}\cos\left(2\pi\frac{lm}{N}\right)}\ . \tag{61}$$

Inverse Fourier Transform $\varkappa = \mathcal{F}^{-1}[\,s\,]$

We rewrite s_m to get

$$\begin{aligned} s_m &= \mathcal{F}[\,\varkappa\,]_m \\ &= \frac{\gamma^2}{\alpha}\frac{1}{(\frac{2}{\alpha}+2k+1) - \underbrace{\left(2\sum\limits_{l=1}^{k}\cos\left(2\pi\dfrac{lm}{N}\right)+1\right)}_{:=z_{k,m}}} \\ &= \frac{\gamma^2}{\alpha(\frac{2}{\alpha}+2k+1)}\left(1-\frac{z_{k,m}}{\frac{2}{\alpha}+2k+1}\right)^{-1} \\ &= \frac{\gamma^2}{\alpha(\frac{2}{\alpha}+2k+1)}\sum_{l=0}^{\infty}\left(\frac{z_{k,m}}{\frac{2}{\alpha}+2k+1}\right)^{l}\ . \end{aligned} \tag{62}$$

Here, we used the geometric series and the fact that $|z_{k,m}| < \frac{2}{\alpha}+2k+1$ for all $\alpha < \infty$.
$z_k \widehat{=} (z_{k,m})_{m=-\infty}^{\infty}$ is a periodic sequence the inverse Fourier transform of which $\zeta_k := \mathcal{F}^{-1}[\,z_k\,]$ yields

$$\begin{aligned} \zeta_{k,n} &= \mathcal{F}^{-1}[\,z_k\,]_n \\ &= \frac{1}{N}\sum_{m=0}^{N-1}z_{k,m}e^{2\pi i\frac{nm}{N}} \\ &= \frac{1}{N}\sum_{m=0}^{N-1}\left\{2\sum_{l=1}^{k}\cos\left(2\pi\frac{lm}{N}\right)+1\right\}e^{2\pi i\frac{nm}{N}} \\ &= \sum_{l=-k}^{k}\frac{1}{N}\sum_{m=0}^{N-1}e^{2\pi i\frac{(n-l)m}{N}} = \sum_{l=-k}^{k}\delta_{nl}\ , \end{aligned} \tag{63}$$

which is the periodic step sequence

$$\zeta_{k,n} = \begin{cases} 1 & \text{if} \quad n \mod N \leq k \quad \text{or} \quad n \mod N \geq N-k \\ 0 & \text{otherwise} \end{cases} . \tag{64}$$

We iteratively define the sequence ζ_k^{*l} of sequences

$$\zeta_k^{*l} := (\zeta_k * \zeta_k^{*(l-1)}) , \quad \zeta_k^{*1} = \zeta_k . \tag{65}$$

Thus, the inverse Fourier transform $\varkappa = \mathcal{F}^{-1}[\,s\,]$ yields

$$\begin{aligned} \varkappa_n &= \mathcal{F}^{-1}[\,s\,]_n = \frac{1}{N}\sum_{m=0}^{N-1} s_m e^{2\pi i \frac{nm}{N}} \\ &= \frac{\gamma^2}{\alpha(\frac{2}{\alpha}+2k+1)} \sum_{l=0}^{\infty} \frac{1}{N} \sum_{m=0}^{N-1} \left(\frac{z_{k,m}}{\frac{2}{\alpha}+2k+1} \right)^l e^{2\pi i \frac{nm}{N}} \\ &= \frac{\gamma^2}{\alpha(\frac{2}{\alpha}+2k+1)} \left\{ \delta_{0n} + \sum_{l=1}^{\infty} \frac{\mathcal{F}^{-1}[\,z_k^l\,]_n}{(\frac{2}{\alpha}+2k+1)^l} \right\} \\ &= \frac{\gamma^2}{\alpha(\frac{2}{\alpha}+2k+1)} \left\{ \delta_{0n} + \sum_{l=1}^{\infty} \frac{\zeta_{k,n}^{*l}}{(\frac{2}{\alpha}+2k+1)^l} \right\} \end{aligned} \tag{66}$$

Hence, the covariance $\varkappa_n$ between two nodes i and $(i+n)$ is an infinite weighted sum of simple sequences.

Monotonicity of ζ_k^{*l}

Let ζ_k be the periodic step sequence

$$\zeta_{k,n} = \begin{cases} 1 & \text{if} \quad n \mod N \leq k \quad \text{or} \quad n \mod N \geq N-k \\ 0 & \text{otherwise} \end{cases} . \tag{67}$$

and let the sequence of sequences ζ_k^{*l} be defined by

$$\zeta_k^{*l} := (\zeta_k * \zeta_k^{*(l-1)}) , \quad \zeta_k^{*1} = \zeta_k . \tag{68}$$

Furthermore, let $k, N \in \mathbb{N}$ and $\delta > 0$ with $2k+1 < N$.
We note that $\zeta_k^{*1} \,\widehat{=}\, \zeta_k$ is symmetric (i.e. invariant under $n \mapsto -n$). Then, by induction, we find that, for all l, ζ_k^{*l} is symmetric:

$$\zeta_{k,-n'}^{*l} = \zeta_{k,n'}^{*l} \tag{69}$$

More importantly, we note that, again by induction, for all l, ζ_k^{*l} is monotonically decreasing in the interval $n \in [0, \frac{N}{2})$, i.e.

$$\zeta_{k,n}^{*l} - \zeta_{k,n+1}^{*l} \geq 0 . \tag{70}$$

Since the sequence $\varkappa$ is a sum of sequences that are symmetric and monotonically decreasing in the interval $n \in [0, \frac{N}{2})$ (compare (66)), we thus conclude that $\varkappa$ itself has these properties.

The Difference $\varkappa_k - \varkappa_{k+1}$

Equation (57) yields the difference $\varkappa_k - \varkappa_{k+1}$:

$$\sum_{l=1}^{k} (\varkappa_{k-l} - \varkappa_{k+1-l}) - 2(\frac{1}{\alpha}+k)(\varkappa_k - \varkappa_{k+1}) + \sum_{l=1}^{k} (\varkappa_{k+l} - \varkappa_{k+1+l}) = 0 \tag{71}$$

$$\Rightarrow \quad \varkappa_0 - \varkappa_k - 2(\frac{1}{\alpha}+k)(\varkappa_k - \varkappa_{k+1}) + \varkappa_{k+1} - \varkappa_{2k+1} = 0 \tag{72}$$

$$\Rightarrow \quad \varkappa_k - \varkappa_{k+1} = \frac{1}{\frac{2}{\alpha}+2k+1} (\varkappa_0 - \varkappa_{2k+1}) \tag{73}$$

Since $\varkappa$ is monotonically decreasing in the interval $n \in [0, \frac{N}{2})$ for $2k+1 < N$, $\varkappa_0 > \varkappa_n$. Importantly, $\varkappa_{2k+1} \neq \varkappa_0$ since we chose k such that it fulfills $2k+1 < N$. Hence,

$$\varkappa_0 - \varkappa_{2k+1} > 0 \quad \Rightarrow \quad \varkappa_k - \varkappa_{k+1} > 0 \; . \tag{74}$$

Conclusion

$\varkappa_n$ is monotonically decreasing for $|n| < \frac{N}{2}$ and the farthermost connected unit is more correlated than the closest connected unit. Hence, connected units are strictly more correlated than unconnected units. Thus, k-ring topologies of this model are always reconstructible.

EVALUATION OF RECONSTRUCTION ERRORS

Receiver operator characteristic (short: ROC or ROC curve) provide a method to visualize and evaluate the quality of binary classifiers. In the manuscript, we use ROC curves to evaluate the discriminative power of correlation thresholding as classifier between links and non-links.

ROC curves and their usefulness to compare classifier properties are discussed extensively in the literature (e.g., compare [2]). For those who are not familiar with the concept we summarize the necessary information regarding our manuscript.

A binary classifier is a functions h which classifies whether a sample $v \in \mathcal{M}$ belongs to a certain class ($h(v) = \text{True}$) or not ($h(v) = \text{False}$). $\mathcal{M}$ is called sample space.

$$h: \; \mathcal{M} \to \{\text{False}, \text{True}\} \tag{75}$$

Let $\mathcal{M}^+ \subseteq \mathcal{M}$ be the set of samples actually belonging to class and let $\mathcal{M}^- \subseteq \mathcal{M}$ be a set of samples not belonging to that class. Let them have cardinalities $N^+ := |\mathcal{M}^+|$ and $N^- := |\mathcal{M}^-|$, so that $\mathcal{M} = \mathcal{M}^+ \cup \mathcal{M}^-$ and $N := |\mathcal{M}| = N^+ + N^-$. Then a perfect classifier has to fulfill the conditions

$$v \in \mathcal{M}^+ \;\Leftrightarrow\; h(v) = \text{True} \tag{76}$$
$$v \in \mathcal{M}^- \;\Leftrightarrow\; h(v) = \text{False} \; . \tag{77}$$

However, real classifiers are usually imperfect; they produce false classifications.
These failures can either be false positive, if a sample is incorrectly classified as a member of the class, or false negative, if a member of the class is not identified as such. Correctly categorized samples constitute true positive or true negative classifications accordingly.
Let $\mathcal{T}^+, \mathcal{T}^-, \mathcal{F}^+, \mathcal{F}^- \subseteq \mathcal{M}$ be the subsets of true positive, true negative, false positive and false negative classifications. Hence,

$$\mathcal{T}^+ \cup \mathcal{F}^- = \mathcal{M}^+ \tag{78}$$
$$\mathcal{T}^- \cup \mathcal{F}^+ = \mathcal{M}^- \; . \tag{79}$$

The fraction of true positive classifications with respect to the overall numbers of positive samples is called true positive rate $t^+ = \frac{|\mathcal{T}^+|}{|\mathcal{M}^+|}$ or *sensitivity* and $f^- = \frac{|\mathcal{F}^-|}{|\mathcal{M}^+|}$ is called false negative rate. True negative rate or *specificity* t^- and the false positive rate f^+ are defined analogously.

Every non-trivial classifier depends on parameters which determine its output. In the manuscript, classifiers depend on one criterion: the correlation threshold. By varying this threshold and measuring sensitivity and specificity, a finger print of performance in f^+-t^+ space is obtained. This finger print is called ROC curve.
Depending on the shape of the curve the quality of the classifier can be extracted visually.
For example, consider the witless random classifier which decides at random with a probability p if a sample is classified positively. For large N^+ the true positive rate is then $t^+ \approx \frac{p \cdot N^+}{N^+} = p$. Same holds for the false positive rate in case of large N^- since $f^+ \approx \frac{p \cdot N^-}{N^-} = p$. Hence, $t^+ = f^+$.
This is why the ROC of every random classifier lies on the identity in f^+-t^+ space.

The ROC curve of an ideal classifier has to intersect the point $(0, 1)$ in f^+-t^+ space because no false positives and false negatives are produced for some criterion value.

When separating two classes by thresholding of a criterion value, the curve start at $(0, 0)$ and end at $(1, 1)$. If both sets can be separated, the classifier is perfect and the ROC has a rectangular shape. The area under the curve will be exactly AUC $= 1$. Otherwise the integral will lead to smaller values.

For each network realization, we computed the correlation matrix C and employed a sliding threshold θ to reconstruct undirected network representations A' in the way discussed above. Plotting the true positive rate $r_t(\theta)$ (the percentage of correctly inferred links) versus the false positive rate $r_f(\theta)$ (the percentage of non-links that where erroneously classified as links) results in the receiver-operator characteristic (ROC) of the decision problem. The area under the curve AUC $= \int r_t \, \mathrm{d}r_f$ is a benchmark for the evaluation of classifiers like discussed above.

[1] C. Gardiner, *Stochastic Methods: A Handbook for the Natural and Social Sciences* (Springer Berlin Heidelberg, 2009) p. 447.

[2] T. Fawcett, Machine learning **31**, 1 (2004).
